# Results of Hospital Sunday, 1895

**Published:** 1895-06-22

**Authors:** 


### Results of Hospital Sunday, 1895.

The Lord Mayor continues to receive cheques sent in response to our special appeal. Some of those who send seem to feel that they are asked to limit their gifts to sums of one pound sterling. This is a misapprehension we should be glad to dispel at once. Already the churches have set an example to individual Londoners of wealth and influence, for Canon Fleming's collection at St. Michael's, Chester Square, amounts to £1,173; and the Rev. C. J. Ridgeway, Christ Church, Lancaster Gate, has sent £1,151, an increase of nearly £100 compared with the collection at his church in 1894. In past years individual donors have sent cheques to the Mansion House for sums varying from £5,000 to £1,000 each, and we earnestly hope that the same splendid generosity may yet be shown by wealthy persons interested in the hospitals.

